# Regenerative endodontic therapy in mature teeth with necrotic pulp and apical periodontitis using two disinfection protocols

**DOI:** 10.1186/s12903-023-02863-w

**Published:** 2023-03-22

**Authors:** Aalaa E. Eldessoky, Mohammed M. Khalefa, Ashraf M. Abu-Seida

**Affiliations:** 1 Department of Endodontics, Faculty of Dental Medicine for Girls, Al-Azhar University, Cairo, Egypt; 2 Department of Surgery, Anesthesiology & Radiology, Faculty of Veterinary Medicine, Cairo University, PO: 12211, Giza, Egypt

**Keywords:** Diode laser, Double antibiotic paste, Periapical pathosis, Regenerative endodontic, Revascularization.

## Abstract

**Objective:**

This study compared the effect of diode laser (DL) 980 nm and double antibiotic paste (DAP) on response of mature teeth with necrotic pulp and apical periodontitis to regenerative endodontic therapy in a dog model.

**Methods:**

Pulp necrosis and periapical pathosis were induced in 40 mature double rooted premolars in four 2-year-old mongrel dogs. These teeth were randomly divided according to disinfection protocol into four equal groups (10 teeth each/20 roots), group I: DAP; group II: DL980 nm; group III: positive control (without treatment) and group IV: negative control (untouched teeth). These groups were further subdivided into 2 subgroups (5 teeth each/10 roots) according to evaluation period, subgroup (A): one month and subgroup (B): three months. Revascularization techniques were performed using bleeding induction and application of platelet rich fibrin (PRF). The coronal cavities were sealed with mineral trioxide aggregate (MTA) and glass ionomer cement. The inflammatory response, vital tissue in-growth, new hard tissue formation and bone resorption were assessed. Statistical analysis was done utilizing ANOVA, Tukey’s post hoc and paired *t* tests.

**Results:**

In both subgroups, there were no significant differences between DAP and DL980 in terms of inflammatory cell count, vital tissue in-growth, new hard tissue formation and bone resorption (P ≤ 0.05).

**Conclusion:**

Diode laser 980 nm can alternate DAP as a disinfection method of the root canal during RET for mature necrotic teeth, therefore it may accelerate regenerative endodontic therapy for both the patient and dentist and allows for RET in a single appointment.

## Background

Nowadays regenerative endodontic therapy (RET) is playing crucial and promising role in treatment of endodontic diseases because the dental pulp complex cells are able to regenerate the damaged tissues to healthy vital tissues [1]. Firstly, RET was evaluated for the management of immature teeth with necrotic pulp [2–9].

Recently, RET has shown promising outcomes in mature teeth [10, 11]. Instead of a mechanical seal made by artificial obturating materials, pulp regeneration offers a biological seal and increases the fracture resistance of the apical part of the root canal [2].

The irreversible loss of the pulp tissue during root canal therapy (RCT) has many drawbacks, such as lack of sensation, crown discoloration and immune mechanism, change of translucency of the tooth and high susceptibility to root fracture. Moreover, RCT is a lengthy and expensive procedure for both the patient and the dental practitioner [10].

Regenerative endodontic treatment in mature teeth could overcome these disadvantages providing a “biological obturation” of the root canal with newly-formed tissues, thereby re-establishing sensory and immune mechanisms. This could probably lower the incidence of flare-ups and might be more fracture resistant than traditionally approach [10].

Effective root canal disinfection is a necessity for RET [8, 10]. Traditional root canal disinfection methods included sodium hypochlorite, mechanical preparation, and calcium hydroxide, but they were inefficient in cases of biofilm-related infection [12]. As a result, additional intracanal drugs were recommended as replacements or supplements for root canal disinfection, like triple antibiotic paste (TAP).

The major difference in RET for teeth with immature necrotic pulps and teeth with mature necrotic pulps is restriction of complete mechanical preparation due to the very thin and fragile dentin root walls. Therefore disinfection of the immature teeth is accomplished with copious irrigation and intracanal medicaments. Complete mechanical debridement is essential to eliminate root canal infection and remove necrotic tissue [10].

Due to the darkening of young, mature teeth caused by minocycline, one of the agents in TAP, that is one of its main disadvantages, double antibiotic paste (DAP) was suggested [12, 13]. DAP at higher doses has a considerable antimicrobial effect but it has detrimental effects on stem cell viability [13].

Due to its antibacterial action, bio-stimulation and improvement of success and longevity utilizing the thermal effect of laser on surrounding tissues, using of laser power has recently gained significant attention in the endodontic practice [14, 15]. Both the patient and the dentist will save the time and prevent the invasive surgical techniques. For dental applications, laser wavelengths typically range between 800 and 1,064 nm [16]. Diode laser use was recommended by a recent study that evaluated its efficacy in maturogenesis of immature teeth with necrotic pulps [17]. However scarce investigations on the diode laser, when used for root canal disinfection in RET of the mature teeth, is available. Therefore, this study compared the effect of diode laser 980 nm and DAP on response of mature teeth with necrotic pulp and apical periodontitis to regenerative endodontic therapy in a dog model.

## Materials and methods

### Ethical approval

The Research Ethics Committee of the Faculty of Dental Medicine for Girls at Al Azhar University, Egypt approved this study (P-EN-21-03). Moreover, all the Animal Research: Reporting of in Vivo Experiments (ARRIVE) guidelines were followed up. All efforts were done to minimize the animals’ pain. All experiments were performed in accordance with relevant guidelines and regulations.

### Animal model

Four 2-year-old mongrel dogs were purchased from Al-Fahad Trading Company for Animals (Abu Rawash, Giza, Egypt) and used in this study. All dogs had a complete set of permanent teeth. The dogs were kept in Department of Surgery, Faculty of Veterinary Medicine, Cairo University, Egypt, in separate cages with optimum ventilation, temperature, hygienic standards, nutritious meals and12 h light/dark cycle. The dogs were given two meals of cocked food (20 g/kg) and milk as well as fresh water ad libitum.

In each dog, 10 premolars were selected to sum 40 teeth constituting 80 root canals. These teeth were divided randomly into four main groups (10 teeth each/20 roots). All groups were represented in each dog. The right side included the positive control and laser groups while the left side included the negative control and DAP groups.

### Sample size calculation

Sample size calculation was done using alpha (α) level of 0.05 (power = 80%) by IBM® SPSS® Sample Power® Release 3.0.1. The calculation was estimated using CDC Epi Info program version 7.2.0.1 (Atlanta, USA). A total sample of a minimum 28 teeth from two dogs (10 each experimental group; and 8 for the control group) was needed based on an estimated mean bacterial count of 1.712 ± 0.848 in experimental group using DL compared to 0.552 ± 0.097 in experimental group using DAP [17].

### Classification of samples

Forty mature premolars were divided into two major experimental groups and two control groups (10 teeth each) according to the disinfection protocol; Group I: DAP, Group II: DL980 nm, group III: positive control group (teeth with induced infection without treatment) and group IV: negative control group (untouched teeth).

These groups were further subdivided into 2 subgroups (5 teeth each/10 roots) according to evaluation period, subgroup (A): one month evaluation period and subgroup (B): three months evaluation period.

### Induction of periapical pathosis

The dogs were given general anesthesia following 12-hour fasting. Atropine sulphate (Atropine sulphate 1%®, ADWIA, Egypt) and Xylazine HCl (Xylaject 2%®, ADWIA, Egypt) were administered subcutaneously and intramuscularly, respectively as premedications. Ketamine HCl (Ketamine hydrochloride®, Rotexmedica Co., Germany) was given intravenously at a dose of 5 mg/kg body weight through a cannula in the cephalic vein to induce anesthesia. General anesthesia was maintained by 2.5% Thiopental sodium (Thiopental sodium®; EIPICO, Egypt) given intravenously at a dose of 25 mg/kg body weight.

Coronal cavity was carried out in all experimental and positive control teeth. Exposing the pulp chamber was made using #2 diamond stone with high-speed handpiece mounted on a portable unit. A sterile file #15 was used to disrupt the pulp. The dog’s supragingival plaque was suspended in sterile saline solution, and a sterile sponge was dipped in the suspension before being put into the pulp chamber. The coronal cavities were filled with cotton for a month after a piece of cotton was inserted into the opening of each canal. For postoperative analgesia, dogs were given soft food and Carprofen tablets (Rimadyl tablet®, Zoetis, USA) orally for 10 days at a dose of 4.4 mg/kg once daily [5–8].

### Preparation of double antibiotic paste

Equivalent amounts of ciprofloxacin (Ciprocin 250 mg Capsule®, EIPICO, Egypt) and metronidazole (Flagyl 250 mg Tab®, Sanofi Aventis, Egypt) were mixed with distilled water to create a diluted concentration of DAP (0.1 mg/mL). An amount of 2.5 g of methyl cellulose powder was mixed with 100 mL of DAP under magnetic stirring to create the homogenous gel that was used in this.

### Root canal preparation and disinfection

Periapical radiographs had been performed under general anesthesia to confirm periapical lesions after the infection phase. All previously affected teeth were accessed and isolated by a rubber dam under aseptic procedures. Using an apex locator, the working length to the anatomical apex was calculated for REP (E- CONNECT, E-PEX Pro, Eighteeth, China). Using the ProTaper Universal system (Dentsply Maillefer, Ballaigues, Switzerland), the root canals were instrumented to the required length up to #F4. Throughout all stages of preparation, 2 mL of 1.5% sodium hypochlorite (NaOCl) (Clorox Co., Egypt) was irrigated into each canal using a 27-gauge side vented needle between files. After a final rinse with 0.9% normal saline (20 mL/canal, 5 min), group I canals were dried with paper points and dressed with DAP. A 20-gauge sterile plastic syringe was used to inject the DAP mixture through the canal. The access cavities were temporarily filled with glass ionomer (Riva Self Cure, SDI Limited, Australia).

On the other hand, group II had mechanical preparation before diode laser disinfection. In order to perform lasing, the root canals were first filled with 2 mL of 1.5% sodium hypochlorite. The laser fiber (980 nm, 2.5w) was then inserted into the canal 1 mm before the apex.

The laser fiber tip (980 nm, 2.5 w) was reinserted 1 mm shorter than its actual working length once the root canals had dried. From the apex to the coronal end and back again, the fiber was moved in a circular manner (5 passes with 5 s in-between where the pass was from the apex to the coronal end). The fiber tip didn’t make contact with the canal’s interior wall [18].

Glass ionomer repair was applied to fill the coronal cavities.

### Treatment modalities

The experimental teeth were re-entered after a month, and the glass ionomer restoration was pulled out using diamond stone under the same general anesthesia and aseptic procedures. For the experimental groups, the following treatment methods were applied to the root canals:

In group I, DAP was removed with copious irrigation with distilled water followed by 6mL of 1.5% NaOCl solution and 17% EDTA solution (Prevest Denpro, Digiana, Jammu, India) (5 mL/canal, 5 min) according to AAE guidelines of irrigation protocol in RET in immature teeth [10, 12]. Distilled water with paper point dryness was used in between irrigations.

In group II, the canal was irrigated with 2 mL of 1.5% NaOCl solution, then activated by diode laser 980 nm at power of 1.5 W with Ton = 10milliseconds; T off = 10milliseconds 50 Hz (50% pulse mode). The 200 micron diameter fiber optic tip was inserted into the canal 1 mm short of its working length and moved helical around the root walls at a speed of 2 millimetres per second. Distilled water with paper point dryness was used in between irrigation. Irrigation with 17% EDTA solution and irradiation with the laser were performed and followed again by 2mL of 1.5% NaOCl irrigation. Final rinsing with distilled water and paper point dryness were performed after the final irrigation with EDTA 17% solution for a minute [19].

### Procedure of pulp regeneration

In order to cause bleeding to fill the canal space, a sterile size #20 hand K-file was inserted 2 mm outside the canal terminal. The blood clot formed over a period of time. The dog’s cephalic vein was used to collect 20 mL of venous blood. The blood sample was immediately centrifuged using a centrifuge (REMI Laboratories, Mumbai, India) at 3000 revolutions per minute for 10 min after being placed into a test tube without an anti-coagulant. Red blood cells formed the lowest layer, followed by PRF in the middle, and platelet-poor plasma at the top. The PRF clot was gently removed using a sterile instrument. The PRF clot was compressed using sterile dry gauze. Using a finger plugger, cut portions of the newly created PRF membrane were inserted gradually in the canal up to the level of the cemento-enamel junction (CEJ). MTA was used to plug orifices and glass ionomer filling was used to close the coronal cavities [6].

### Bio-stimulation technique

In both groups I and II, 980-nm diode laser (Pioon, China) was used for laser irradiation for 15 s at 0.5 W power and a tip diameter of 10 mm. With the tip of the laser handpiece held at a distance of about 10 mm from the mucosa, the soft tissues covering the mesial and distal apices of the tooth were exposed to laser radiation from the buccal and lingual sides [20].

### Histological evaluation

The animals were euthanized using an anesthetic overdose (Thiopental sodium) then, their jaws were separated, and bone segments, including the experimental and control teeth were removed. Blocks were fixed in 10% buffered formalin solution at a fixative to tooth mass ratio of 1:5 for two weeks. The blocks were decalcified by 17% EDTA solution for 120 days.

The decalcified samples were prepared, sectioned into buccolingual sections of 6 *u*m thickness and embedded in paraffin blocks. Sections were stained with hematoxylin and eosin dye for histopathology as follows:

#### Inflammation

For each slide, fields with well-preserved tissue, good architecture, no artifacts and intense inflammatory cells infiltration were selected.

Total inflammatory cell number was counted using image analysis software (Image J, USA) as follows:

In order to exclude other undesirable structures, the color-coding threshold was modified to identify the perimeter of the entire spectrum of inflammatory cells. Prior to calculation, a binary threshold of the selected color-coded inflammatory cells was performed.

Inflammation was evaluated according to previous studies [2–8] as follows: score 0: Absence or very few cells, score 1 (mild): less than 10 cells on average, score 2 (moderate): between 10 and 25 cells and score 3 (severe): more than 25 cells on average.

#### Vital tissue in-growth inside the canal space

It was assessed according to previous studies [2–8] as follows: score 0: no tissue ingrowth inside the root canal space, score 1: evidence of tissue in growth in the apical third of the canal space, score 2: evidence of tissue in growth in the middle third of the canal space and score 3: evidence of tissue in growth in the cervical third of the canal space.

#### New hard tissue formation

It was examined according to previous studies [2–8] as follows:

##### Qualitative analysis

Criteria for histologic identification of hard structure included; dentin (presence of dentinal tubules), cementum (presence of cementocyte-like cells adherent to dentin), bone (presence of Haversian canals with uniformly distributed osteocyte-like cells) and periodontal ligament (presence of Sharpey’s fibers bridging cementum and bone).

##### Quantitative analysis

This was evaluated as follows: score 0: absence of new hard tissue, score 1: partial formation of new hard tissue and score 2: complete formation of new hard tissue.

#### Bone resorption

It was evaluated as follows: score 0: absence of signs of resorption and score 1: presence of signs of active resorption including osteoclasts, Howship’s lacunae and areas of resorption.

### Statistical analysis

Data management and statistical analysis were performed using the Statistical Package for Social Sciences (SPSS) version 20. Regarding the number of inflammatory cells (normally distributed numeric variable), one way analysis of variance (ANOVA) test was used for comparisons between groups, followed by Bonferroni’s post hoc test. Comparison between both observation periods was performed using paired t test. Kruskal Wallis test was used for comparisons between groups with respect to percent of reduction (non-parametric numeric variable). Regarding scores for vital tissue, hard tissue formation and bone resorption, Kruskal Wallis test was used for comparisons between groups, whereas comparison between both observation periods was performed using Wilcoxon signed rank test.

The percent change was calculated by the formula: value after-value before/ value before X100.

All P-values were two-sided. P-values ≤ 0.05 were considered significant.

## Results

### Findings of the inflammation

**Fig. 1:**
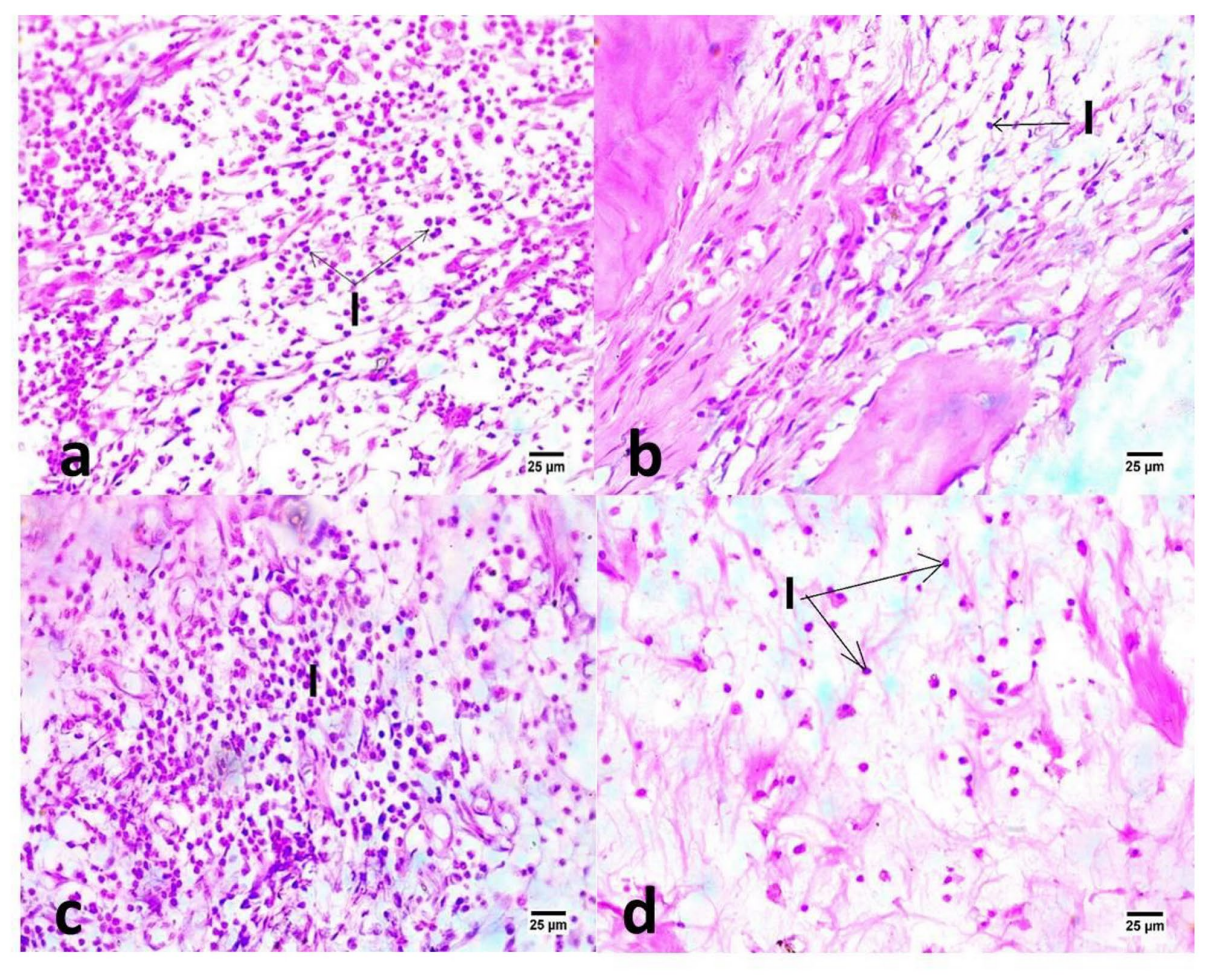
Photomicrographs of samples in different groups: DAP (a), DL (b), positive control (c) and negative control (d) after one month evaluation period showing various numbers of mononuclear inflammatory cells (I & arrows) between tooth root and bone alveoli (400X, H&E).

In subgroup A (one month), the highest mean number of inflammatory cells was recorded in positive control group, followed by group I, then group II, with the least number recorded in negative control group (Fig. 1). The difference between control and experimental groups was statistically significant (P ≤ 0.05). Post hoc test revealed no significant difference between groups I and II (P > 0.05) as shown in (Table 1).

**Table 1:** Comparison of f inflammatory cells count in different groups and subgroups (ANOVA test)

Subgroups	Groups	Mean	SD	P-value
Subgroup A (One month)	Group I (Double antibiotic paste)	237.10^b^	48.13	0.000*
	Group II (Diode laser disinfection)	192.90^b^	62.81	
	Group III (Positive control group)	545.30^a^	103.20	
	Group IV (Negative control group)	86.00^c^	20.68	
Subgroup B (Three months)	Group I (Double antibiotic paste)	168.50^y^	43.93	0.000*
	Group II ((Diode laser disinfection)	146.00^y^	29.19	
	Positive control group	1213.70^x^	208.26	
	Negative control group	74.80^z^	21.99	

*Significant at P ≤ 0.05. Post hoc test: Within the same comparison, means sharing the same superscript letter are not significantly different

**Fig. 2:**
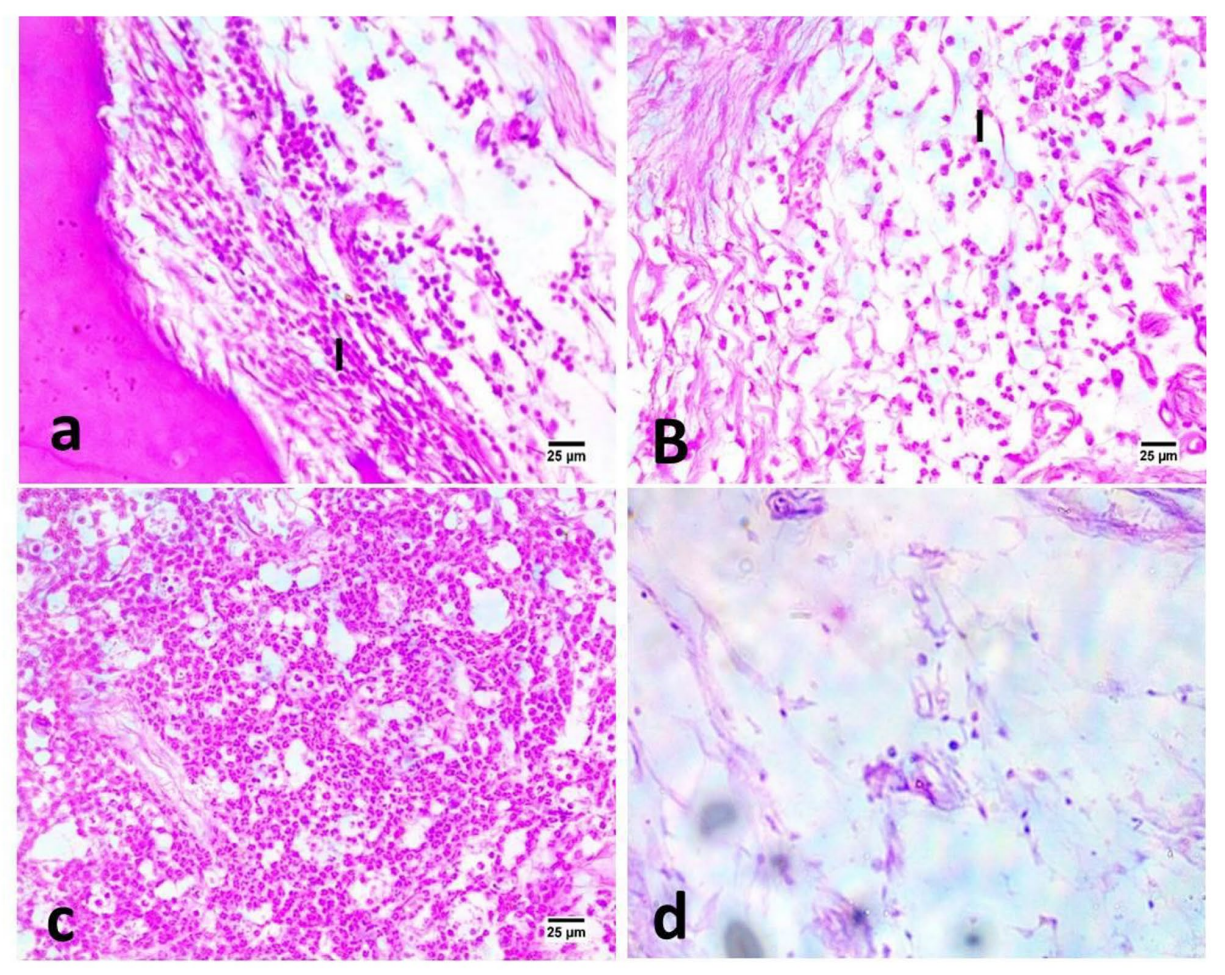
Photomicrographs of samples in different groups: DAP (a), DL (b), positive control (c) and negative control (d) after three months evaluation period showing various numbers of mononuclear inflammatory cells (I) between tooth root and bone alveoli (400X, H&E).

In subgroup B (Three months), the highest mean number of inflammatory cells was recorded in positive control group, followed by group I, then group II, with the least number recorded in negative control group (Fig. 2). The difference between control and experimental groups was statistically significant (P ≤ 0.05). Post hoc test revealed no significant difference between groups I and II (P ≥ 0.05).

Regarding the percentage of change from the first to the 3rd month, the greatest median of % decrease was recorded in group I (-34.5%), followed by group II (-20.62%). Negative control showed a median value (0%), whereas positive control group showed a mean percentage increase (103.68%).

Intra group comparison is summarized in (Table 2).

**Table 2:** Intragroup comparison of inflammatory cells count (effect of time) by paired *t* test

Groups	Subgroups	Mean	SD	P-value
Group I (Double antibiotic paste)	Subgroup A	237.10	48.13	0.01*
	Subgroup B	168.50	43.93	
Group II (Diode laser)	Subgroup A	192.90	62.81	0.05*
	Subgroup B	146.00	29.19	0.00*
Group III (Positive control group)	Subgroup A	545.30	103.20	
	Subgroup B	1213.70	208.26	
Group IV (Negative control group)	Subgroup A	86.00	20.68	0.29 ns
	Subgroup B	74.80	21.99	

*Significant at P ≤ 0.05

In groups I (DAP) and II (DL), the mean value of inflammatory cells decreased significantly after 3 months (P ≤ 0.05). While in positive control group, the mean value of inflammatory cells increased significantly after 3 months (P ≤ 0.05). In negative control group, the mean value of inflammatory cells decreased insignificantly after 3 months (P ≥ 0.05).

### Nature and extent of vital tissue in-growth in the pulp space

**Fig. 3:**
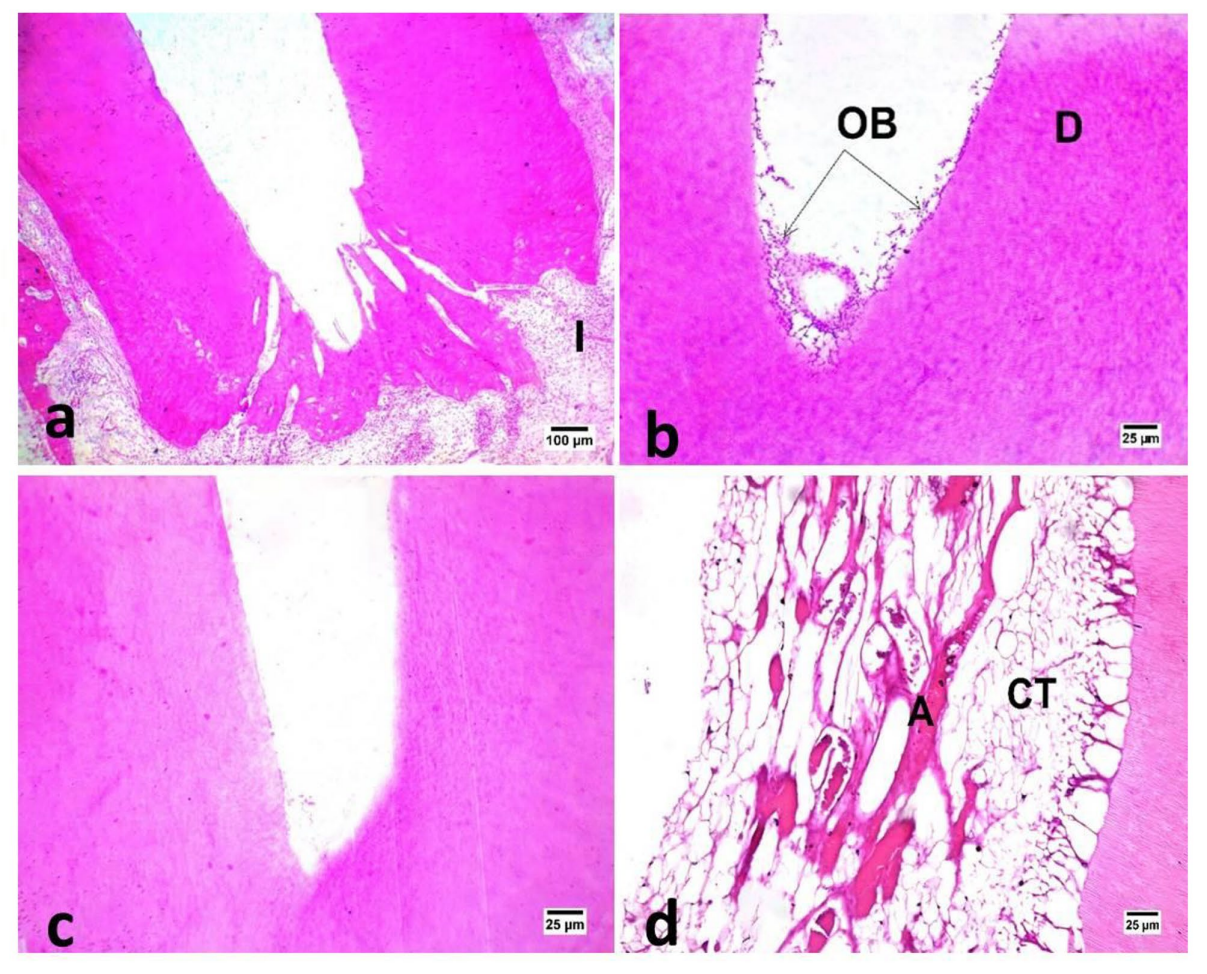
Photomicrographs of samples in different groups: DL (b), positive control (c) and negative control (d) after one month evaluation period showing vital tissue in-growth inside the pulp space (400X, H&E). Notice the multiple layers of odontoblasts (OD) in inner surface of dentin (D) in DL group and the fibrous connective tissue (CT) with angiogenic activity (A) in negative control group. Various numbers of mononuclear inflammatory cells (I) between tooth root and bone alveoli in DAP (a) (100X, H&E).

**Fig. 4:**
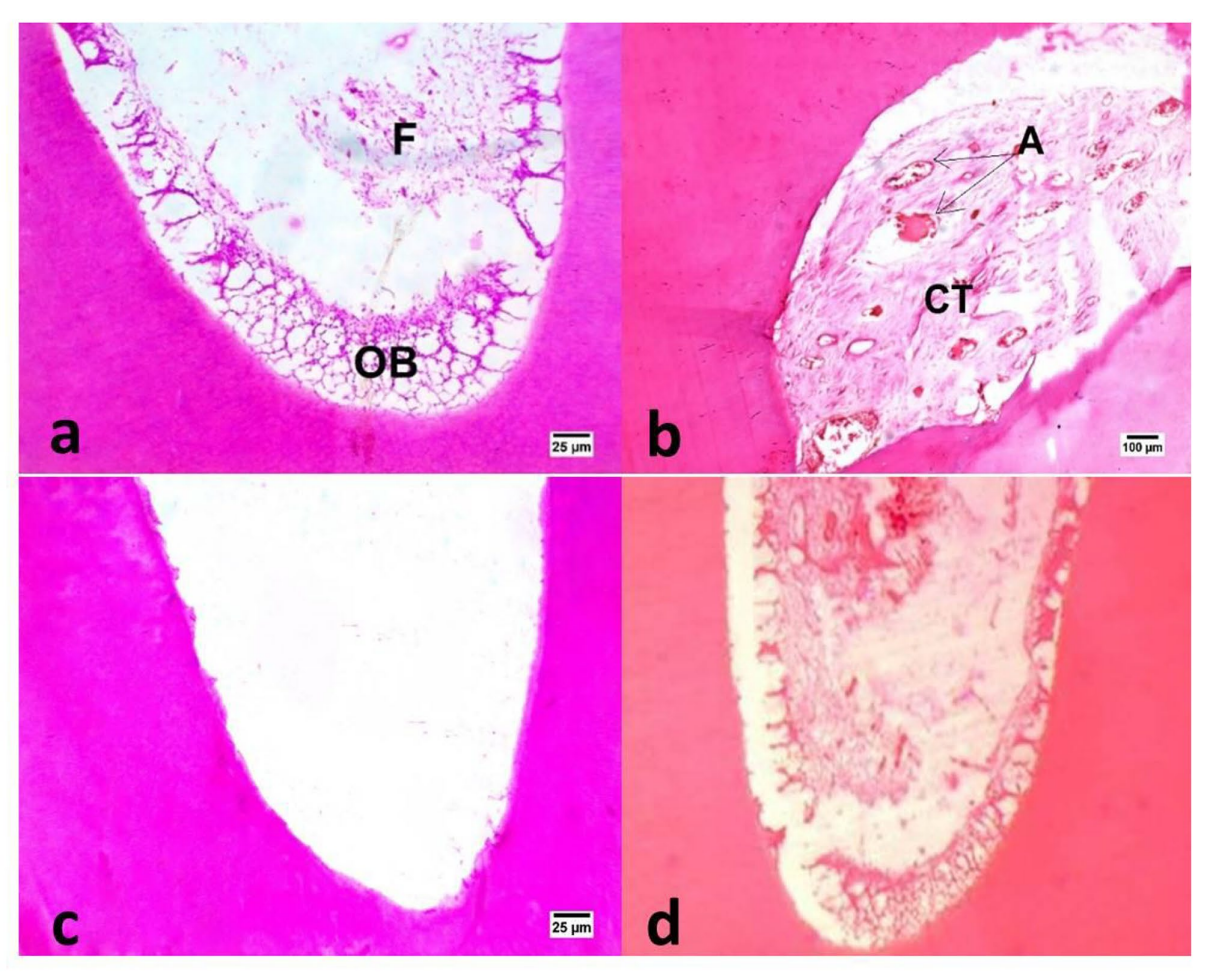
Photomicrographs of samples in different groups: DAP (a), positive control (c) and negative control (d) after three months evaluation period showing vital tissue in-growth inside the pulp space (400X, H&E). Notice vital tissue in-growth inside the apical third of the canal space in both DAP (b) and DL groups showing multiple layers of odontoblasts (OD), fibrous connective tissue (F & CT) and angiogenic activity (A & arrows) (100X, H&E).

The regenerated tissue inside the canal space was a newly formed tissue that resembled periodontal connective tissue, with varying degrees of inflammatory cell infiltration and a low to moderate number of blood vessels at the central portion that were engorged by red blood cells (Figs. 3 and 4).

In subgroup A, negative control recorded a significantly higher value than other all groups (P ≤ 0.05) as shown in (Table 3; Fig. 3).

**Table 3:** Descriptive statistics and comparison of vital tissue scores in different groups (Kruskal Wallis test) and within the same group (Wilcoxon signed rank test).

Groups	Vital tissue	Subgroup A (One month)	Subgroup B (Three months)	P-value within group
Group I (Double antibiotic paste)	Median	0.00^b^	3.00^a^	.046*
	Min	0.00	0.00	
	Max	0.00	3.00	
Group II (Diode laser)	Median	0.00^b^	3.00^a^	.046*
	Min	0.00	0.00	
	Max	0.00	3.00	
Group III (Positive control group**)**	Median	0.00^b^	0.00^b^	1 ns
	Min	0.00	0.00	
	Max	0.00	0.00	
Group IV (Negative control group**)**	Median	3.00^a^	3.00^a^	1 ns
	Min	3.00	3.00	
	Max	3.00	3.00	
**P-value between groups**		.000*	.006*	

In subgroup B, groups I and II recorded a median score (3), whereas positive control group recorded median score (0) and negative control group recorded a median score (3) as shown in (Table 3; Fig. 4). The difference between positive control group and other all groups was statistically significant (P ≤ 0.05). Post hoc test revealed no significant difference between group I, group II and negative control group (P ≥ 0.05).

In groups I and II, a significantly higher median score was recorded at 3 months than at one month (P ≤ 0.05). In positive control group, a median score (0), ranging from (0 to 0) was recorded in both observation times. While in negative control group, a median score (3), ranging from (3 to 3) was recorded in both observation times.

### New hard tissue formation

In subgroup A, all groups recorded median score (0). In subgroup B, groups I and II recorded a median score (0), ranging from (0 to 1), whereas positive and negative control groups recorded median score (0), ranging from (0 to 0). The difference between groups was not statistically significant (P ≥ 0.05) as shown in (Table 4).

**Table 4:** Descriptive statistics and comparison of hard tissue formation scores in different groups (Kruskal Wallis test) and within the same group (Wilcoxon signed rank test)

Groups	Hard tissue formation	Subgroup A (One month)	Subgroup B (Three months)	P-value within group
Group I (Double antibiotic paste)	Median	0.00	0.00	0.317 ns
	Min	0.00	0.00	
	Max	0.00	1.00	
Group II (Diode laser)	Median	0.00	0.00	0.317 ns
	Min	0.00	0.00	
	Max	0.00	1.00	
Group III (Positive control group**)**	Median	0.00	0.00	1ns
	Min	0.00	0.00	
	Max	0.00	0.00	
Group IV (Negative control group)	Median	0.00	0.00	1ns
	Min	0.00	0.00	
	Max	0.00	0.00	
**P-value between groups**		1 ns	0.55 ns	

Significance level at P ≤ 0.05, ns = non-significant

**Fig. 5:**
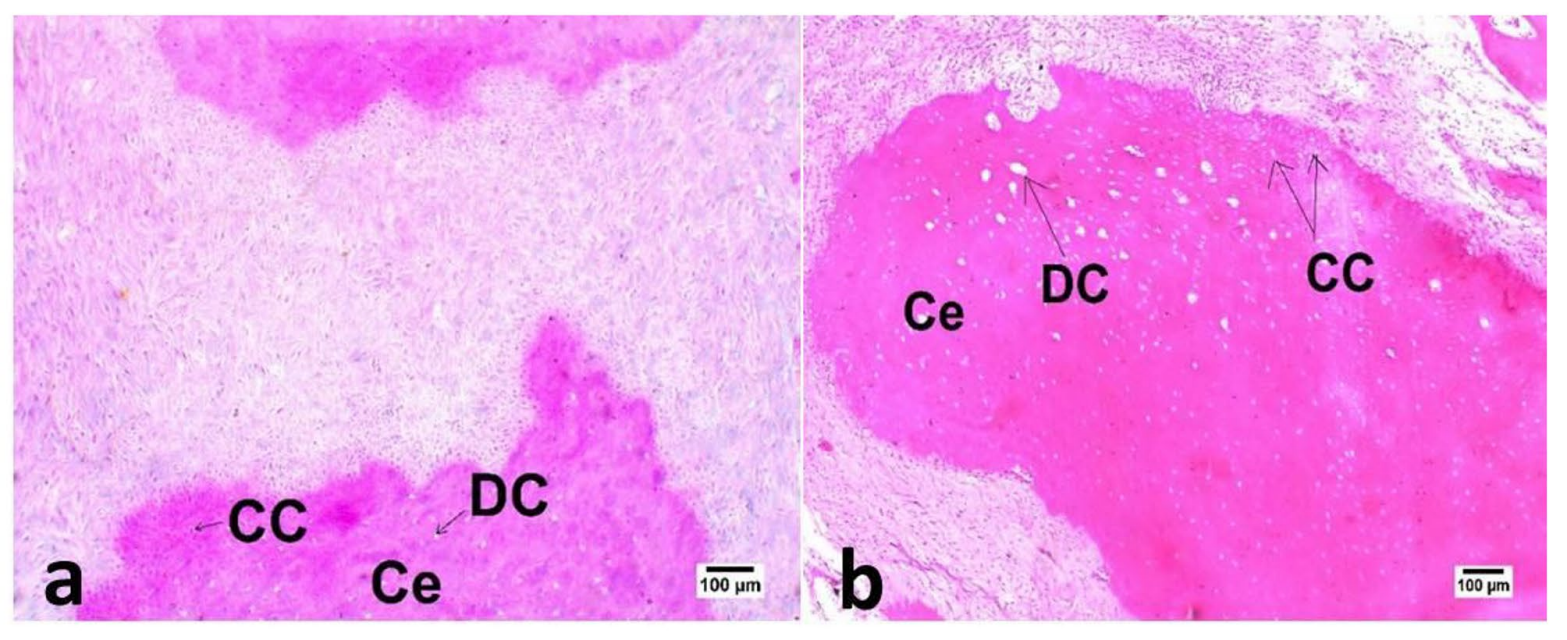
Photomicrographs of samples in DAP (a) and DL (b) groups after three months evaluation period showing apical new hard tissue formation that resemble cementum like tissue (Ce) with presence of cementocyte like cells (CC) and degenerated cementocyte like cells (DC) (100X, H&E).

Qualitative assessment of certain samples of the experimental groups’ newly created hard tissue in subgroup B revealed cementum-like tissue that laid down on the root’s outer surface and was protected by a thin layer of cementoid tissue. Cementocyte- and cementoblast-like cells were also found (Fig. 5).

### Bone resorption

No signs of bone resorption were recorded in all groups and subgroups. There were no Howships lacunae observed in the qualitative analysis.

## Discussion

In last decade, RET of necrotic teeth gained more attention due to its ability to overcome many difficulties of the conventional endodontic therapy like risk of fracture, multiple visits, and expensive costs [10, 11]. The present study compared DAT and DL980 as methods of root canal disinfection during RET and concluded that both methods have nearly similar efficacy. Therefore, DL can replace DAP and save more time for both dentist and patient. Moreover, the known disadvantages of DAP, like the emergence of bacterial resistance, can be avoided by disinfecting with DL980 during RET.

Like many previous studies, dogs were chosen as an animal model for this study because of their similar apical healing to humans in a shorter amount of time [5]. To increase the total number of samples for a reliable statistical analysis, the double rooted premolars were selected. Moreover, premolars were chosen because their canals are of proper size [5, 6, 21].

Because of its strong antibacterial actions, ability to preserve stem cells, and capability to release growth factors which are necessary in RET, 1.5% NaOCl solution was used for irrigation in this investigation [22]. In group I, a total amount of 14mL 1.5% NaOCl solution was used in the first visit as 2mL of 1.5% NaOCl solution in-between each file (7 files were used) then we used 6 mL of the same solution in the second visit to sum 20 mL of 1.5% NaOCl solution according to AAE guidelines of irrigation protocol in RET in immature teeth [10, 12]. In addition, we used 17% EDTA solution on the superficial dentin layer as conditioning due to its demineralizing effect with the release of growth factors and elimination of the loosely attached smear layer [4, 10, 23].

ProTaper Universal rotary files were used to instrument canals because their convex triangular cross-section that decreases friction between the blade and the canal wall and increased cutting effectiveness. Additionally, their design makes cutting easier, removes dirt from the canal, and prevents unintentional screwing. The apical preparation of the canals was completed with an F4 ProTaper Universal file to ensure that the root canals were properly cleaned and shaped [24].

In the current study, we used 0.1 mg/mL DAP for one month because 1 mg/mL DAP in an earlier experiment had no significant changes in direct antibacterial activity [10]. DAP also significantly affects microorganisms and negatively affects stem cell survival at this high concentration [13]. Also, 5 mg/mL of DAP or higher for a month resulted in significantly higher residual antibiofilm effect than pretreating dentin with the same concentrations for one week [24]. Although DAP is difficult to use at 0.1 mg/mL, methyl cellulose was used as a vehicle to create a consistency appropriate for clinical use and to extend the duration of the therapeutic agent [25, 26].

In the current study, PRF was used due to its three-dimensional design, autologous origin, and bioactive chemicals that promote regeneration through stimulation of multipotent stem cell mineralizing differentiation ability [7, 27]. Due to its biocompatibility and strong mechanical and sealing properties, the MTA was used as orifice plug [2, 7, 28].

Following endodontic therapy, final coronal restoration was carried out to prevent reinfection inside the canals. The success rate of root canal therapy is better for teeth with high-quality restorations than for teeth with poor restorations [29]. Therefore, glass ionomer was applied for coronal restoration in this study.

Diode laser was applied in this investigation due to its efficacy in lowering intra-canal bacterial counts and penetrating 500 microns into dentin, low cost and smaller size than other types of lasers [19, 30, 31]. In the current work, intracanal irradiation was performed in a pulsed manner to decrease the risk of thermal injury to the external root surface, periodontal tissues, or underlying bone, which minimizes postoperative pain and enhances periapical healing [14, 15].

The laser fiber was moved in a circular manner from the apex to the coronal end and back again to make sure the laser covered the whole interior wall of the canal. To prevent melting the dentin and spreading the thermal impact to the region of the periodontal ligaments, the internal canal wall was not touched with the fiber tip. Also, the fiber tip was kept clean in order to create a powerful laser beam [19].

In the present study, we used bio-stimulation to enhance the regeneration. Using bio-stimulation technique as low-level laser treatment stimulates regeneration processes without significantly increasing body temperature because it enhances cellular metabolism, protein expression, cell migration, proliferation, and differentiation [20].

After one and three months, all experimental groups differed significantly from the negative control group in terms of the inflammatory scores. These results may be explained by the periradicular tissues’ inflammatory response to the treatment procedures that were carried out, which was then increased by the immune system’s response to the previously created infection [32]. The mean inflammatory scores were lower in each experimental group after 3 months. This shows that the removal of the etiological variables induced gradual healing of the periapical lesion. In contrast, the positive control group experienced a significantly higher level of inflammation as a result of the generated infection’s development and absence of therapy [33].

Regarding the tissue in-growth within the root canal space, the type of regenerated tissue resembled the periodontal connective tissue, with inflammatory cell infiltration and blood vessels. Similar findings were recorded before [5]. In contrast, pulp-like fibrous connective tissue with calcified bone islands and cementum, and without an odontoblastic layer was seen in another study [34].

In subgroup A, small number of samples in the experimental groups exhibited restricted development of vital tissue inside the canal space. Depending on the quantity and aggressiveness of the bacteria present as well as the host’s immune system, healing may occur in the infected canals after RET [28]. In contrast, the positive control group exhibited no vital tissue in-growth due to the absence of blood clot (scaffold) and presence of severe inflammation that inhibit tissues regeneration.

In both subgroups, there was no significant difference between the groups in term of the creation of new hard tissue. These findings are consistent with the results of an earlier research [34]. In subgroup B, the newly formed hard tissue resembled cementum, which was distinguished by inclusion of cementocyte-like cells.

Both DAP and DL980 groups exhibited nearly similar partly new hard tissue due to the homogeneity of the repair mechanisms across both groups. The absence of vital tissue in-growth, which is necessary for hard tissue deposition, may explain why the positive control group did not exhibit hard tissue deposition. These results agreed with the results of a prior study [35].

In the present work, there was no evidence of bone resorption. This finding contrasts with the findings of a previous study that found bone resorption in some revascularization samples up to three months after surgery as a result of an inflammatory response accompanied by the release of inflammatory mediators like prostaglandins and interleukins [11].

Hmud et al. discovered that the creation of water vapor caused by a 980-nm diode laser might cause cavitation on a water-based media. Therefore, breakdown of the irrigation solution can be accelerated that improves disinfection. The bubble pressure in the root canal can tear cell membranes, dislodge biofilms of bacteria, and remove debris and the smear layer [36].

Several future studies are recommended such as comparison between the antibacterial effects of diode laser980nm and 1.5% NaOCl in regeneration, using of diode laser980nm to disinfect the cases of failed root canal therapy, and assessment of the antibacterial effects of diode laser980nm on various microbial endodontic species.

## Conclusions

Diode laser 980 nm can alternate DAP as a disinfection method of the root canal during RET for mature necrotic teeth, therefore it may accelerate RET for both the patient and dentist and allows for RET in a single appointment.

## Data Availability

The datasets used and/or analysed during the current study available from the corresponding author on reasonable request.

## Abbreviations

DL980: Diode laser 980
PRF: platelet rich fibrin
MTA: mineral trioxide aggregate
RET: Regenerative endodontic therapy
TAP: Triple antibiotic paste
NaOCl: sodium hypochlorite
CEJ: Cementoenamel joint
